# Trends﻿ in the prevalence of adult overweight and obesity in Australia, and its association with geographic remoteness

**DOI:** 10.1038/s41598-021-90750-1

**Published:** 2021-05-31

**Authors:** Syed Afroz Keramat, Khorshed Alam, Mohammed Khaled Al-Hanawi, Jeff Gow, Stuart J. H. Biddle, Rubayyat Hashmi

**Affiliations:** 1 Economics Discipline, Social Science School, Khulna University, Khulna, 9208 Bangladesh; 2 School of Business, University of Southern Queensland, Toowoomba, QLD 4350 Australia; 3 Centre for Health Research, University of Southern Queensland, Toowoomba, QLD 4350 Australia; 4 Department of Health Services and Hospital Administration, Faculty of Economics and Administration, King Abdulaziz University, Jeddah, Saudi Arabia; 5 School of Accounting, Economics, and Finance, University of KwaZulu-Natal, Durban, 4000 South Africa; 6 Health Economics Research Group, King Abdulaziz University, Jeddah, Saudi Arabia

**Keywords:** Diseases, Risk factors, Signs and symptoms

## Abstract

The prevalence of overweight and obesity has been increasing globally and has become a significant public health concern in Australia in the two past decades. This study explores the most recent national prevalence and trends of adult overweight and obesity in Australia. It will also investigate geographic remoteness as a potential risk factor for an individual being overweight or obese in adulthood. A retrospective longitudinal study that utilised 14 successive waves (wave 6 through 19) of a nationally representative linked individual-level survey. Data was obtained from the Household, Income and Labour Dynamics in Australia survey. The data on 199,675 observations from 26,713 individuals aged ≥ 15 years over the period 2006 to 2019 was analysed. Random-effects logit model was employed to estimate the association between geographic remoteness and the risk of excessive weight gain. The results reveal that the prevalence of overweight, obesity and combined overweight and obesity among Australian adults in 2019 were 34%, 26% and 60%, respectively. The analysis shows that the prevalence of overweight and obesity varies by geographic remoteness. Adults from regional city urban (OR 1.53, 95% CI 1.16–2.03) and rural areas (OR 1.32, 95% CI 1.18–1.47) were more likely to be obese compared with their counterparts from major city urban areas. The results also show that adults living in major city urban areas, regional city urban areas, and regional city rural areas in Australia were 1.53 (OR 1.53, 95% CI 1.16–2.03), 1.32 (OR 1.32, 95% CI 1.18–1.47), and 1.18 (OR 1.18, 95% CI 1.08–1.29) times more likely to be overweight compared with their counterparts from major city urban areas in Australia. Substantial geographic variation in the prevalence of overweight and obesity exists among Australian adults and appears to be increasing. Public health measures should focus on contextual obesogenic factors and behavioural characteristics to curb the rising prevalence of adult obesity.

## Introduction

Obesity has been defined as the accumulation of excessive body fat that has adverse health effects. In 2016, 13% (over 650 million) of adults aged ≥ 18 years were obese worldwide^1^. In 2017–18, the combined rate of adult overweight and obesity was 67% in Australia^2^. It is predicted that the Australian adult obesity rate alone will reach 35% by 2025. It is also projected that the rate of severe obesity (Body Mass Index [BMI] ≥ 35) will reach 13% by 2025 from just 5% in 1995^3^.

Obesity is an emerging public health concern in Australia^4^. Overweight and obesity together is the second leading risk factor contributing 8.4% of the total disease burden in Australia, behind tobacco use^5^. Overweight and obesity are linked with an increased risk of non-communicable diseases (NCDs), such as cancers, cardiovascular diseases, musculoskeletal disorders, kidney disease, diabetes, asthma, dementia, sleep apnea^1,6,7^, and long-term health conditions or disability^8^. Further, obesity contributes substantially to labour productivity losses in the form of high absenteeism^9^, presenteeism^10^ and low job satisfaction^11^ in the workplace. Therefore, the future direct (health burden) and indirect (productivity loss) costs will more likely increase with obesity’s rising prevalence in Australian society.

Analyses of geographic disparity in the prevalence of NCDs are essential for public health intervention as they identify the conditions’ above-average prevalence or ‘hot spots’. Geographical disparity also exists in the prevalence of adult obesity. Discordant results have been found in the literature regarding the association between geographic remoteness and obesity in developed countries^12–18^. A recent United States (US) study confirmed that substantial geographic differences exist in obesity prevalence among Asian Americans^15^. Four empirical studies conducted in the US and European countries indicate that living in rural settings is positively associated with overweight and obesity^13,14,17,18^. However, a study of 10 European countries provides evidence that the prevalence of obesity does not vary between rural and urban settings^16^. Moreover, some studies have documented within-country (e.g., state-level or region-level) variation in overweight and obesity prevalence in developed countries, such as the US, Canada and Finland^19–21^.

The prevalence of overweight and obesity in all age groups has risen dramatically over the last three decades in Australia. The Australian Institute of Health and Welfare reports the adulthood obesity rate at the national level^4^; however, little is known about geographic remoteness, within-country variations, such as remoteness and urban–rural settings. Furthermore, previous studies have not examined geographic remoteness and individual characteristics in tandem when determining the risk factors of obesity. Limited efforts have been made to explore the association between geographic remoteness and obesity using longitudinal data. Given the high prevalence in the trends of adulthood obesity in Australia and the large geographic distances and contexts experienced, it would be prudent to investigate the longitudinal association between geographic remoteness and increased risk of being obese. Therefore, the present study aims to document the prevalence of adult overweight and obesity in Australia and report the longitudinal association between geographic remoteness with the risk of being overweight and obese.

The present study is novel because it captures the association between geographic remoteness and adulthood obesity along with the distribution and comparison of obesity prevalence within Australian cities and rural–urban areas. The findings will be valuable in supporting public health initiatives to halt the obesity epidemic.

## Methods

### Data source and sample selection

The present study data were extracted from the nationally representative Household, Income and Labour Dynamics in Australia (HILDA) survey. HILDA is a large-scale household-based longitudinal survey that collects data annually from over 13,000 individuals within over 7000 households. Since 2001, it has collected information on various aspects of the individuals’ lives, including income, wealth, labour status, fertility, health, education, skills and more. The survey collects data from individuals aged 15 years and above in the household using a combination of self-completed questionnaires and face-to-face and telephone interviews by trained interviewers. The details of the HILDA survey design have been described elsewhere^22^.

Information concerning BMI, the primary variable of interest, is available from wave 6 onwards in the HILDA survey. Therefore, this study considered data from wave 6 through 19 of the HILDA survey, spanning from 2006 to 2019. The entire HILDA cohort (waves 6 through 19) consists of 297,120 person-year observations. However, a total of 97,445 observations were dropped due to non-response (73,952) and non-matching (23,493) for the self-completion paper questionnaire (SCQ). After excluding non-response and non-matching observations from the original sample, the working sample comprises of 199,675 yearly observations from 26,713 individuals at (up to) fourteen different time points. The present analysis utilised supplied responded person SCQ weights to retain the national representativeness of the study sample. After using supplied responded person SCQ weights, the estimated population size ranged from 15,115,558 (corresponding unweighted sample size of 11,716 individuals in wave 6) to 19,109,375 (corresponding unweighted sample size of 16,150 in wave 19). Year-wise unweighted sample and weighted population size have been provided in Table 1 of Appendix A. A detailed description of the HILDA survey weights has been outlined elsewhere^23^. The present study conducted a missing observation analysis and found that nearly 5% of responses were missing for the variable, BMI (please refer to Table 2 of Appendix A). This study utilised the last observation carried forward (LOCF) method after controlling individual for imputing missing responses to produce conservative estimates.

### Outcome variable

The present study is primarily interested in adult overweight and obesity, measured through an internationally standardised BMI measure. This study used self-reported height and weight to compute BMI using the formula, weight (kg)/height^2^ (metre). To define the participant’s weight status, this study categorised BMI into underweight (BMI < 18.50), healthy weight (BMI 18.50 to < 25), overweight (BMI 25 to < 30) and obese (BMI ≥ 30) following the World Health Organization (WHO) cut off points^1^. BMI was further recoded into binary form (‘healthy’ weight versus overweight or obese) as the two possible outcomes for the multivariate regression analysis.

### Exposure variable

The primary exposure variable investigated in this study is geographic remoteness, measured through remoteness (major city, regional city, and remote areas), and place of residence (urban and rural settings). These two variables were used to construct the variable, geographic remoteness. One of the significant geographical units of analysis in the HILDA survey is remoteness. Remoteness is measured through the Australian Statistical Geography Standard (ASGS) Remoteness Structure, which divides remoteness into five groups: major cities, inner regional, outer regional, remote, and very remote based on the road distances that people have to travel to access key services^24^. This study collapsed remoteness into two categories: major cities and regional cities (merging inner regional, outer regional, remote and very remote areas). Another geographical unit of measurement in HILDA is residence, a binary variable of urban and rural settings. This measure is quite different from the remoteness area measure. During the survey, each individual’s household was assigned according to the 2001 Census Collection District (CD). Population counts from the 2001 Census were then used to classify CDs as urban or rural settings^25^. Using these two variables (remoteness and place of residence), this study formed a new mutually exclusive variable, geographic remoteness. This study categorised geographic remoteness into four groups: major city urban areas, major city rural areas, regional city urban areas, and regional city rural areas.

### Covariates

This study considered covariates based on previous research on the risk factors of adult obesity in Australia^26,27^. The socio-demographic covariates included age (15–24, 25–54, 55–64 and 65 or above years), gender (male and female), education (year 12 or below, professional qualification and university qualification), civil status (single, married/living together, and divorced/widow/separated), household income quintile (quintile 1 referring to the lowest income group and quintile 5 referring to the highest income group), labour force status (employed, unemployed and not in the labour force), and ethnicity (not of indigenous origin and Aboriginal or Torres Strait Islander [ATSI] or both). Behavioural characteristics included alcohol consumption (former or non-drinker or current drinker) and smoking cigarettes or tobacco products (former/non-smoker or current smoker).

### Estimation strategy

An unbalanced panel data set was constructed through the individual’s record’s linkage, with most participants included in the analytic sample up to fourteen times (wave 6 through 19). The study participants’ characteristics have been summarised in the form of frequency (n) and percentages (%) with 95% confidence intervals (CIs). The prevalence of obesity is reported in the form of percentages (%) by geographic remoteness. The bivariate association between the main variables of interests and covariates with the outcome variable were checked through chi-square tests. All the predictors were entered into the final model only when a predictor was significant at a 5% or less statistical significance level in the bivariate analysis. Two separate regressions were fitted to check the association between overweight and obesity with geographic remoteness adjusted for age, gender, education, civil status, household income, labour force status, ethnicity, smoking status, and alcohol consumption.

To estimate the association between BMI and geographic remoteness, random-effects logit models were deployed. For ease of interpretation of the results, adjusted odds ratio (aOR) with 95% CIs were reported. This study assessed all multivariate models at the 5% level of statistical significance and performed all statistical analyses using Stata 16 (StataCorp LLC).

### Ethics approval

This paper uses unit record data from Household, Income and Labour Dynamics in Australia Survey (HILDA) conducted by the Australian Government Department of Social Services (DSS). However, the findings and views reported in this paper are those of the authors and should not be attributed to the Australian Government, DSS, or any of DSS contractors or partners. https://doi.org/10.26193/OFRKRH, ADA Dataverse, V2. This study did not require ethical approval as the analysis used only de-identified existing unit record data from the HILDA survey. However, the authors completed and signed the Confidentiality Deed Poll and sent it to NCLD (ncldresearch@dss.gov.au) and ADA (ada@anu.edu.au) before the data applications’ approval. Therefore, the datasets analysed and/or generated during the current study are subject to the signed confidentiality deed.

## Results

Table 1 describes the pooled BMI classification, geographic remoteness, socio-demographic and behavioural characteristics for the 199,675 person-year observations. The pooled prevalence of overweight and obesity was nearly 34% and 24%, respectively. Among the participants, 50% were in the age group 25 to 54 years, 53% were female, 59% were married, 25% had university qualifications, 33% were not in the labour force, 97% were not of Indigenous origin, 18% were current smoker, and 82% consumed alcohol. A large majority of the respondents lived in major city urban areas (65%) in Australia, followed by regional city rural areas (22%).

**Figure 1:**
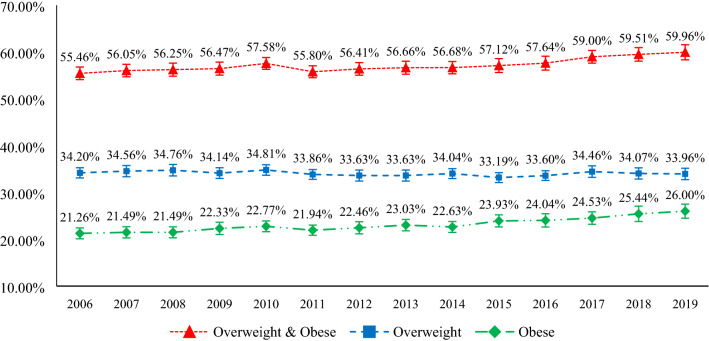
Overweight and obesity trends in Australia, 2006–2019.

Figure 1 displays the trends in overweight, obesity, combined rates of adult overweight and obesity from 2006 to 2019 in Australia. The prevalence of overweight and obesity in Australia were 34% and 26%, respectively, in 2019. Figure 1 also shows that the prevalence of combined overweight and obesity rate increased by five percentage points (55% in 2006 to 60% in 2019), and obesity alone increased by five percentage points (from 21% in 2006 to 26% in 2019) over the 14-year study period.

**Figure 2:**
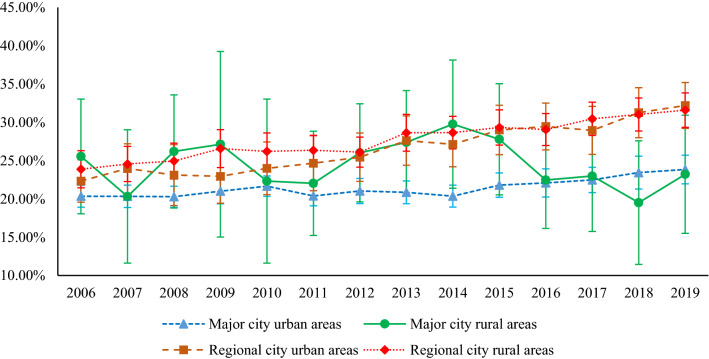
Trends in the prevalence of obesity by geographic remoteness in Australia, 2006–2019.

Figure 2 demonstrates the trends in the prevalence of obesity among Australian adults from 2006 to 2019 by geographic remoteness. A high variation in the prevalence of obesity regarding major and regional cities has been observed, along with an increasing trend in obesity from regional city urban and rural areas. Figure 2 also reveals that rates of adult obesity in major city urban area, major city rural area, regional city urban area, and regional city rural area were 24%, 23%, 32%, and 32%, respectively, in 2019. Further, it shows that obesity rates ranged from 22 to 32% in regional city urban areas and 24% to 32% in regional city rural areas over the study period (2006–2019).

Table 2 displays the results of the adjusted multivariate regression analyses for the longitudinal association between geographic remoteness, overweight, and obesity. The results showed that adults living in major city urban areas, regional city urban areas, and regional city rural areas in Australia were 1.53 (OR 1.53, 95% CI 1.16–2.03), 1.32 (OR 1.32, 95% CI 1.18–1.47), and 1.18 (OR 1.18, 95% CI 1.08–1.29) times more likely to be overweight compared with their counterparts from major cities urban areas in Australia (model 1). The results also showed that geographic remoteness is positively associated with a higher risk of being obese. The results revealed that the likelihood of being obese were 1.49 (OR 1.49, 95% CI 1.16–1.92) and 1.31 (OR 1.31, 95% CI 1.07–1.60) times higher among adults living in regional city urban and regional city rural areas of Australia, respectively, compared with their peers living in major city urban areas (model 2).

**Table 1:** Background characteristics of the study participants.

Variables	n	% (95% CI)
**Body mass index (BMI)**		
Underweight (< 18.50)	5,355	2.68 (2.61–2.75)
Healthy weight (18.50 to < 25.00)	78,330	39.23 (39.01–39.44)
Overweight (25.00 to < 30.00)	68,358	34.23 (34.03–34.44)
Obesity (≥ 30)	47,632	23.85 (23.67–24.04)
**Age**		
15–24 years	34,365	17.21 (17.05–17.38)
25–54 years	100,079	50.12 (49.90–50.34)
55–64 years	29,344	14.70 (14.54–14.85)
≥ 65 years	35,887	17.97 (17.80–18.14)
**Gender**		
Male	93,455	46.80 (46.58–47.02)
Female	106,220	53.20 (52.98–53.42)
**Education**		
Year 12 or below	88,795	44.47 (44.25–44.69)
Professional qualifications	61,703	30.90 (30.70–31.10)
University qualifications	49,177	24.63 (24.44–24.82)
**Civil status**		
Single	46,335	23.21 (23.02–23.39)
Married/living together	118,494	59.34 (59.13–59.56)
Divorced/Widow/Separated	34,846	17.45 (17.29–17.62)
**Household income quintile**		
Quintile 1 (lowest)	39,935	20 (19.83–20.18)
Quintile 2	39,935	20 (19.83–20.18)
Quintile 3	39,935	20 (19.83–20.18)
Quintile 4	39,935	20 (19.83–20.18)
Quintile 5 (highest)	39,935	20 (19.83–20.18)
**Labour force status**		
Employed	126,686	63.45 (63.23–63.66)
Unemployed	7,479	3.75 (3.66–3.83)
Not in the labor force	65,510	32.81 (32.60–33.01)
**Ethnicity**		
Non-indigenous	194,582	97.45 (97.38–97.52)
Aboriginal/Torres Strait Islander	5,093	2.55 (2.48–2.62)
**Geographic remoteness**		
Major city urban areas	129,473	64.84 (64.63–65.05)
Major city rural areas	2,256	1.13 (1.08–1.18)
Regional city urban areas	23,433	11.74 (11.60–11.88)
Regional city rural areas	44,513	22.29 (22.11–22.48)
**Smoking status**		
Former/non-smoker	162,937	81.60 (81.43–81.77)
Current smoker	36,738	18.40 (18.23–18.57)
**Alcohol consumption**		
Former/non-drinker	38,315	19.19 (19.02–19.36)
Current drinker	161,360	80.81 (80.64–80.98)

**Table 2:** Multivariate analysis for the adjusted associations between overweight and obesity with geographic remoteness.

Variables	Model 1	Model 2
	Overweight versus healthy weight	Obesity versus healthy weight
**Geographic remoteness**		
Major city urban areas (ref)		
Major city rural areas	**1.53 (1.16–2.03), 0.003**	1.63 (0.85–3.13), 0.14
Regional city urban areas	**1.32 (1.18–1.47), < 0.001**	**1.49 (1.16–1.92), 0.002**
Regional city rural areas	**1.18 (1.08–1.29), < 0.001**	**1.31 (1.07–1.60), 0.01**
**Age**		
15–24 years (ref)		
25–54 years	**3.73 (3.40–4.08), < 0.001**	**6.32 (5.16–7.74), < 0.001**
55–64 years	**6.46 (5.73–7.29), < 0.001**	**9.61 (7.29–12.67), < 0.001**
≥ 65 years	**7.26 (6.30–8.36), < 0.001**	**10.06 (7.30–13.87), < 0.001**
**Gender**		
Male (ref)		
Female	**0.26 (0.24–0.30), < 0.001**	**0.53 (0.43–0.66), < 0.001**
**Education**		
Year 12 or below (ref)		
Professional qualifications	**1.70 (1.54–1.88), < 0.001**	**3.27 (2.63–4.07), < 0.001**
University qualifications	1.10 (0.99–1.24), 0.09	**0.64 (0.50–0.81), < 0.001**
**Civil Status**		
Single (ref)		
Married/living together	**2.42 (2.22–2.65), < 0.001**	**5.04 (4.14–6.13), < 0.001**
Divorced/Widow/Separated	**2.39 (2.10–2.72), < 0.001**	**5.18 (3.91–6.84), < 0.001**
**Household income quintile**		
Quintile 1 (lowest)	**0.71 (0.65–0.77), < 0.001**	**0.35 (0.28–0.43), < 0.001**
Quintile 2	**0.71 (0.65–0.76), < 0.001**	**0.52 (0.43–0.63), < 0.001**
Quintile 3	**0.80 (0.75–0.86), < 0.001**	**0.60 (0.49–0.72), < 0.001**
Quintile 4	**0.91 (0.86–0.97), 0.01**	0.86 (0.72–1.03), 0.09
**Quintile 5 (highest) (ref)**		
Labor force status		
Employed (ref)		
Unemployed	**0.89 (0.80–0.99), 0.04**	1.25 (0.97–1.60), 0.08
Not in the labor force	**0.93 (0.87–0.99), 0.03**	**1.51 (1.29–1.77), < 0.001**
**Ethnicity**		
Non-indigenous (ref)		
Aboriginal/Torres Strait Islander	**4.02 (2.88–5.60), < 0.001**	**14.34 (6.67–30.84), < 0.001**
**Smoking status**		
Former/non-smoker (ref)		
Current smoker	**1.34 (1.25–1.43), < 0.001**	**0.52 (0.44–0.62), < 0.001**
**Alcohol consumption**		
Former/non-drinker (ref)		
Current drinker	**0.77 (0.71–0.83), < 0.001**	**1.58 (1.35–1.84), < 0.001**

*ref* Reference.

Values in bold are statistically significant at *p* < 0.05.

## Discussion

The present study firstly observed geographic disparities in the prevalence of adult overweight in Australia. Secondly, it checked the association between geographic remoteness and adult obesity. The results have provided further evidence that the prevalence of adult obesity across Australia has been increasing over time and that large geographic disparities exist in the prevalence of obesity. A substantial geographic difference in the prevalence, along with an increasing trend in obesity, has been observed over the 14-year study period. The prevalence of overweight and obesity combined in the present study is 60%, which is slightly lower than the national estimates. According to the National Health Survey (NHS) conducted every five years by the Australian Bureau of Statistics (ABS), the prevalence of overweight and obesity combined was 67% in 2017–18 among Australians aged 18 and over. One of the possible reasons for the underreported overweight and obesity rates could be that the present study considered adults aged 15 years and over. The prevalence of overweight and obesity is usually low in the younger age group. Sensitivity analysis was performed and it was found that the combined prevalence of overweight and obesity was 63% among Australians aged 18 years or over. The present study findings suggest that the prevalence of obesity in Australia has increased from 21% in 2006 to 26% in 2019. Further, the results revealed that the prevalence of adult obesity in regional city urban areas (22% to 32%) and regional city rural areas (24% to 32%) had increased sharply over the 14-year study period.

The study findings strongly support the hypothesis that there is a positive association between remoteness and excess body weight. The results reveal that the prevalence of adult obesity is greater in both regional city urban and rural areas compared with major city urban areas in Australia. The findings have been corroborated by a past study from Australia, which reported that living in regional towns and remote regions was associated with a higher probability of being obese^26^. The present study finding supports the hypothesis that geographic disparity persists in the prevalence of adult obesity in Australia. An earlier US-based study also supports this finding, where substantial geographical differences (by US census division and region) in the prevalence of obesity have been reported^19^. Further, within-country variation in the prevalence of obesity has also been observed in the Canadian and Finnish populations^20,21^. Moreover, this finding is in line with the conclusion of two studies conducted in Norway and the US, wherein the risk of being obese was higher among rural dwellers than urban counterparts^13,14^. However, this finding contradicts a study of 10 European countries in which no significant association between excess body weight and place of residence was detected^16^.

The geographic disparity in obesity might be due to more risky behaviours, such as poor diet, excessive alcohol consumption and physical inactivity among regional residents than their peers in major cities. A potential explanation for the variation in the prevalence of obesity across remoteness might be due to ethnicity. For example, the higher presence of Aboriginal people in a particular geographical area or a higher proportion of people born overseas in some geographic locations^21^. Furthermore, obesogenic factors, such as social pressure and limited opportunities for physical activities, could be potential risk factors for obesity. Besides, substantial geographic disparities in the prevalence of adult obesity could be attributed to socio-economic position, lifestyle, culture and genetic factors^12^.

The study findings have some important public health implications since they revealed a statistically significant association between geographic remoteness and obesity. Australian federal, state, territory and local governments can play an important role in formulating and implementing health-related policies for maintaining a healthy weight, especially targeting adults living in regional cities and remote areas. Mass media for creating awareness, educational campaigns, and workplace health promotion could help reduce obesity level^28^. Public health intervention should focus on improving contextual obesogenic factors associated with geographic remotenesses, such as creating opportunities for physical activity and access to healthy foods, especially in rural areas. It should be recognised that obesity is associated with many factors and that this area is complex and will require taking a whole systems approach^29^.

The study findings have several strengths. Much of the information regarding the prevalence and trend in adult obesity in Australia comes from studies conducted at the national level. However, very little is known about the obesity rate in small geographical units such as regional cities or remote areas, and urban or rural locations in Australia. The present study is one of the largest epidemiological undertakings on geographical variation in adult obesity in a nationwide sample of the Australian adult population. Further, this study has considered a new geographical characteristic, geographic remoteness (by merging remoteness [major city versus regional city and remote areas] and place of residence [urban versus rural]) to check the geographical disparity in adult overweight and obesity. Another strength of this study is the considerable sample size (n = 199,675), which enables getting the precise estimates of the association between geographic remoteness and obesity, as well as the nationally representative prevalence of obesity.

This study has some limitations that should be considered. This research cannot identify causal pathways between geographic remoteness and obesity due to the unbalanced longitudinal research design. Control over the selection of covariates was also limited as several relevant factors, such as dietary habits, exercise patterns, sedentary behaviours, sleep patterns and quality and the presence of comorbidity, were not considered due to the unavailability of data. Another potential limitation is self-reported BMI to measure overweight and obesity that might underestimate the true prevalence as people systematically underreport weight and over-report height, resulting in lower BMI estimates^30,31^. Besides, there is a possibility of misreporting of height and weight that differed by the geographic remoteness. In taking these limitations into account, the findings suggest that future research should focus on a prospective longitudinal study to explain further the role of geographic remoteness concerning excessive weight gain over time.

## Conclusions

This study has revealed the trend and obesity risk among Australian adults by examining individual and geographical characteristics using a nationally representative data set. It was revealed that substantial variance persists in the prevalence of adult obesity across geographic areas in Australia. Geographic remoteness is positively associated with a higher likelihood of obesity. Estimates from random-effects logit models confirm that living in both regional city urban areas and rural areas were associated with higher odds of being obese compared with living in major city urban areas. The risk of being overweight has been found to be higher among adults living in major city rural areas, regional city urban, and rural areas than their peers living in major city urban areas. Geographically targeted public health interventions and health education for creating awareness and promoting a healthier lifestyle might help combat the obesity epidemic in Australia. This study contributes to the limited literature regarding geographical variation in adult overweight and obesity in Australia.

## Supplementary information

Supplementary Information.

## Data Availability

The data used for the study was collected from the Melbourne Institute of Applied Economic and Social Research. There are some restrictions on this data and it is not available to the public. Those interested in accessing this data should contact the Melbourne Institute of Applied Economic and Social Research, The University of Melbourne, VIC 3010, Australia.

## Abbreviations

ATSI: Aboriginal or Torres Strait Islander
BMI: Body mass index
HILDA: Household, income and labour dynamics in Australia survey
OR: Odds ratios
WHO: World Health Organization
