# Supplementary material for: Trends﻿ in the prevalence of adult overweight and obesity in Australia, and its association with geographic remoteness
Source: Sci Rep. 2021 May 31;11:11320. doi: 10.1038/s41598-021-90750-1 (PMC8166878; doi:10.1038/s41598-021-90750-1)
Supplement: Supplementary file 1 — Supplementary Information. [file 41598_2021_90750_MOESM1_ESM.docx]

**Appendix A**

**Table 1.** Person-year observations across study years

| Year | Waves | N | Weighted population size | % | Cumulative % |
| --- | --- | --- | --- | --- | --- |
| 2006 | 6 | 11,716 | 15,115,558 | 5.87 | 5.87 |
| 2007 | 7 | 11,381 | 15,452,496 | 5.70 | 11.57 |
| 2008 | 8 | 11,194 | 15,768,595 | 5.61 | 17.17 |
| 2009 | 9 | 11,563 | 16,139,759 | 5.79 | 22.96 |
| 2010 | 10 | 12,052 | 16,442,982 | 6.04 | 29.00 |
| 2011 | 11 | 15,366 | 16,711,584 | 7.70 | 36.70 |
| 2012 | 12 | 15,389 | 17,013,844 | 7.71 | 44.40 |
| 2013 | 13 | 15,360 | 17,332,799 | 7.69 | 52.10 |
| 2014 | 14 | 15,595 | 17,559,353 | 7.81 | 59.91 |
| 2015 | 15 | 15,513 | 17,855,647 | 7.77 | 67.67 |
| 2016 | 16 | 16,253 | 18,180,579 | 8.14 | 75.81 |
| 2017 | 17 | 16,194 | 18,540,853 | 8.11 | 83.92 |
| 2018 | 18 | 15,949 | 18,860,088 | 7.99 | 91.91 |
| 2019 | 19 | 16,150 | 19,109,375 | 8.09 | 100 |
|  | Total | 199,675 |  | 100 | 100 |

**Table 2.** Missing observation analysis

| Variable | Missing | Available | % missing |
| --- | --- | --- | --- |
| BMI | 10,122 | 189,553 | 5.34% |
| Remoteness | 21 | 199,654 | 0.01% |
| Civil status | 8 | 199,667 | 0.00% |
| Ethnicity | 76 | 199,599 | 0.04% |
| Education | 94 | 199,581 | 0.05% |
| Smoking status | 2,324 | 197,351 | 1.18% |
| Alcohol consumption | 2,412 | 197,263 | 1.22% |
